# Genome-Wide Identification and Functional Analysis of DNA Methylation-Related Genes in *Sophora tonkinensis* Under Cadmium and Drought Stress

**DOI:** 10.3390/plants15030396

**Published:** 2026-01-28

**Authors:** Fan Wei, Shuangshuang Qin, Linxuan Li, Zhu Qiao, Danfeng Tang, Guili Wei, Yang Lin, Ying Liang

**Affiliations:** 1 Guangxi Key Laboratory of Medicinal Resources Protection and Genetic Improvement/Guangxi Engineering Research Center of TCM Resource Intelligent Creation, National Center for TCM Inheritance and Innovation, Guangxi Botanical Garden of Medicinal Plants, Nanning 530023, China; 2 National Engineering Research Center for Southwest Endangered Medicinal Materials Resources Development, Guangxi Botanical Garden of Medicinal Plants, Nanning 530023, China

**Keywords:** *Sophora tonkinensis*, DNA methylation, cytosine-5 DNA methyltransferase, DNA demethylase, cadmium, drought stress

## Abstract

*Sophora tonkinensis* is a valuable medicinal plant whose cultivation is constrained by drought and cadmium (Cd) contamination. DNA methylation, mediated by cytosine-5 DNA methyltransferases (C5-MTases) and DNA demethylases (dMTases), contributes to plant stress response; however, these gene families have remained uncharacterized in *S. tonkinensis*. Here, we identified 12 methylation-related genes (four *StC5-MTases* and eight *StdMTases*) and analyzed their phylogeny, duplication, promoter cis-elements, and expression patterns under Cd exposure and drought/rehydration. Most duplicated pairs showed Ka/Ks < 1, consistent with purifying selection. *StCMT1* and *StMET2* were induced by both Cd and drought stress but declined after rehydration, whereas *StROS1b* and *StROSlike3* responded rapidly to both stresses. Heterologous overexpression in *Nicotiana benthamiana* improved growth under Cd stress for *StCMT1* lines and under PEG-induced osmotic stress for *StROSlike3* lines, as reflected by plant height and whole-plant fresh weight. Together, these findings establish a genome-wide resource for DNA methylation machinery in *S. tonkinensis* and provide candidate genes for investigating epigenetic regulation of abiotic stress adaptation.

## 1. Introduction

DNA methylation is a key epigenetic mechanism that regulates plant growth and development by modulating gene expression, through either transcriptional repression or activation. It plays crucial roles in processes such as seed germination, vernalization, flowering, and endosperm development [1,2,3,4]. Beyond development, DNA methylation is also essential for plant responses to various abiotic stresses, including drought, salinity, heat, and heavy-metal exposure. Under stress, dynamic changes in methylation can reshape the expression of stress-responsive genes, supporting acclimation and survival [5,6]. Recent studies emphasize the role of epigenetic regulation, particularly DNA methylation, in plant adaptation to toxic metals like cadmium (Cd), with potential implications for stress memory and breeding for tolerance [7,8]. In recent years, genome-wide methylome profiling, such as whole-genome bisulfite sequencing (WGBS), combined with transcriptome analysis, has become a key method for investigating stress-responsive epigenetic regulation. Integrated methylome–transcriptome studies under drought have revealed differentially methylated regions linked to stress-responsive genes and pathways, potentially suggesting the involvement of DNA methylation in transcriptional reprogramming during stress acclimation [9,10].

The methylation landscape is dynamically maintained by two antagonistic enzyme groups: cytosine-5 DNA methyltransferases (C5-MTases), which add methyl groups to cytosines, and DNA demethylases (dMTases), which actively remove these marks. Plant C5-MTases are commonly classified into four subfamilies based on domain composition and biological function: MET1, which maintains CG methylation; CMT, which predominantly maintains CHG and CHH methylation; DRM, which catalyzes de novo methylation via RNA-directed DNA methylation (RdDM); and DNMT2-like enzymes, whose roles in plant DNA methylation remain less clear [11,12]. Active DNA demethylation is mainly mediated by DEMETER (DME)-family 5-methylcytosine DNA glycosylases, including ROS1 (Repressor of Silencing 1), DML2, and DML3, which initiate base-excision repair to remove methylated cytosines [13,14].

C5-MTase and dMTase gene families have been systematically cataloged in many plant species and often show lineage-specific patterns. Examples include the model *Arabidopsis thaliana* [15], and diverse crops or medicinal plants such as *Arachis hypogaea* [16], *Solanum melongena* [17,18], *Camellia sinensis* [19], *Actinidia chinensis* [20], *Dendrobium officinale* [21], *Solanum tuberosum* [22], *Cyclocarya paliurus* [23], *Amaranthus cruentus* [24], and *Theobroma cacao* [25].

*Sophora tonkinensis* Gagnep. is a woody medicinal plant native to the karst regions of Guangxi, Yunnan, and Guizhou province in southwestern China, a habitat characterized by periodic drought and heterogeneous rocky soils [26]. This environment has also been associated with naturally high background Cd levels and a significant risk of Cd pollution. In particular, drought and Cd contamination can severely reduce growth, yield, and medicinal quality of *S. tonkinensis*. Notably, these stresses may co-occur in the field, and water deficit can alter Cd mobility/bioavailability and plant uptake, thereby modulating Cd toxicity and plant performance under combined stress conditions [27,28].

Recent field evidence further supports that Cd-contaminated soils can compromise the productivity and quality of *S. tonkinensis*, whereas soil amendments (e.g., co-application of biochar and organic fertilizer) can reduce Cd accumulation in the rhizosphere and plant tissues and increase the contents of major bioactive alkaloids such as matrine and oxymatrine, likely through improving soil physicochemical properties and reshaping the rhizosphere bacterial community [29,30]. In addition, moderate drought stress has been reported to promote the accumulation of effective constituents in medicinal plants, whereas excessive drought can substantially inhibit the growth of *S. tonkinensis*, ultimately reducing yield and medicinal quality [31,32].

As a woody species adapted to such stress-prone environments, *S. tonkinensis* may possess intrinsic stress-buffering mechanisms distinct from those of more sensitive crops, including a robust antioxidant defense system, tight regulation of ion homeostasis, and potentially long-term epigenetic regulatory processes (e.g., DNA methylation). Although direct cross-species comparisons were not performed here, this ecological and agronomic background supports the rationale for using *S. tonkinensis* to investigate stress adaptation mechanisms in a perennial medicinal plant context.

Despite the recognized importance of DNA methylation in stress adaptation, the C5-MTase and dMTase repertoires of *S. tonkinensis* have not been characterized at the genome-wide level, and their transcriptional dynamics under drought and Cd stress remain unclear. To elucidate how DNA methylation may contribute to stress adaptation in *S. tonkinensis*, this study aimed to: (1) identify C5-MTase and dMTase genes in the *S. tonkinensis* genome; (2) infer their evolutionary relationships and potential functional conservation using phylogenetic analyses; (3) quantify the transcript levels of these genes in roots under controlled Cd exposure as well as drought and subsequent rehydration; and (4) functionally validate two key candidate genes (*StCMT1* and *StROSlike3*) through heterologous overexpression in *Nicotiana benthamiana* and subsequent phenotypic analysis under Cd or drought stress. Overall, our results provide a molecular framework for understanding DNA methylation machinery in *S. tonkinensis* and support future epigenetics-informed breeding strategies.

## 2. Results

### 2.1. Genome-Wide Identification and Classification of C5-MTases and dMTases in S. tonkinensis

Based on analyses of conserved domains, motifs, gene structures, and phylogenetic relationships, four StC5-MTase genes and eight StdMTase genes were identified in the *S. tonkinensis* genome (Table 1). StC5-MTase proteins ranged from 384 amino acids (StDNMT2) to 1519 amino acids (StMET2), with an average length of 1057 amino acids. Their molecular weights ranged from 43.73 to 170.94 kDa (mean, 119.40 kDa), and their theoretical pI values ranged from 5.47 to 6.23. Subcellular localization prediction suggested that all StC5-MTases localize to the nucleus. StdMTases showed greater variation in protein size (280–976 amino acids) and biochemical properties, with pI values from 4.70 to 9.66 and molecular weights from 31.62 to 109.33 kDa. Subcellular localization prediction suggested that most StdMTases are nuclear, whereas WoLF PSORT predicted chloroplast targeting for StROS1a, StROSlike1, and StROSlike4, and Plant-mPLoc supported chloroplast localization only for StROSlike4 (Supplemental Table S1). These in silico predictions require experimental validation (e.g., GFP-fusion assays).

Sequence similarity analysis indicated that *StMET1* and *StMET2* were the most similar pair among StC5-MTases. Within StdMTases, *StROSlike2*/*StROSlike3* and *StROS1b*/*StROS1c* showed notably higher similarity than other members (Figure S1). These patterns are consistent with duplication events contributing to the expansion of both gene families.

### 2.2. Evolutionary and Structural Features of StC5-MTases and StdMTases

#### 2.2.1. Phylogenetic Relationships of C5-MTases and dMTases Across Representative Plant Species

**Figure 1:**
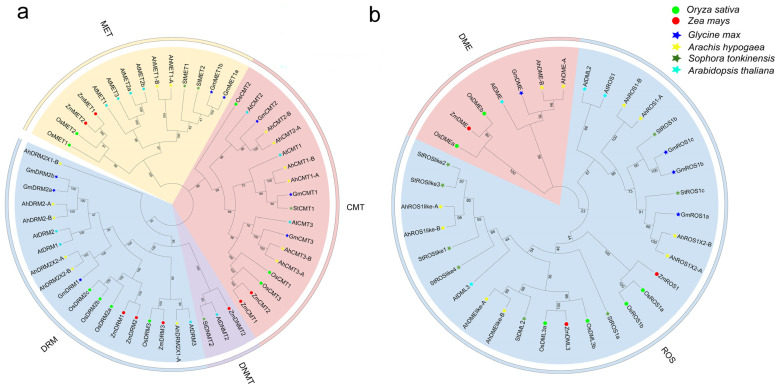
Phylogenetic analysis of the StC5-MTases and StdMTases. (**a**) Neighbor-Joining (NJ) tree of the StC5-MTases. (**b**) NJ tree of the StdMTases. Os, *Oryza sativa*; Zm, *Zea mays*; Gm, *Glycine max*; Ah, *Arachis hypogaea*; At, *Arabidopsis thaliana*.

To clarify the evolutionary relationships of C5-MTase genes in *S. tonkinensis*, an unrooted Neighbor-Joining (NJ) tree was constructed using 54 C5-MTase protein sequences from six plant species. Based on tree topology, C5-MTases clustered into four clades (MET, DNMT, CMT, and DRM) (Figure 1a). DRM and DNMT formed a closely related group, whereas MET and CMT formed separate clusters. The DRM clade contained the most members, while the DNMT clade was the smallest (three genes across three species). In *S. tonkinensis*, one gene was assigned to each of the CMT and DNMT clades, and two genes to the MET clade. Consistent with its taxonomic position, StC5-MTases were most similar to homologs from the legumes *Glycine max* and *Arachis hypogaea*. Moreover, C5-MTases from dicots (*S. tonkinensis*, *A. hypogaea*, *G. max*, and *Arabidopsis thaliana*) were clearly separated from those of monocots (*Oryza sativa* and *Zea mays*). To examine dMTase evolution, an unrooted NJ tree was built using 35 full-length dMTase proteins from the same six species. The dMTases separated into two major groups (Figure 1b). ROS and DML members showed closer relationships to each other than to the DME clade, resulting in a ROS/DML grouping distinct from DME. The DME subfamily proteins formed a compact cluster. Similar to C5-MTases, dMTases from monocots (*O. sativa* and *Z. mays*) formed a distinct branch relative to dicots. The Maximum Likelihood (ML) topology was largely consistent with the NJ results and supported the same subfamily classifications (Figure S2).

#### 2.2.2. Conserved Domains and Motifs of StC5-MTases and StdMTases

**Figure 2:**
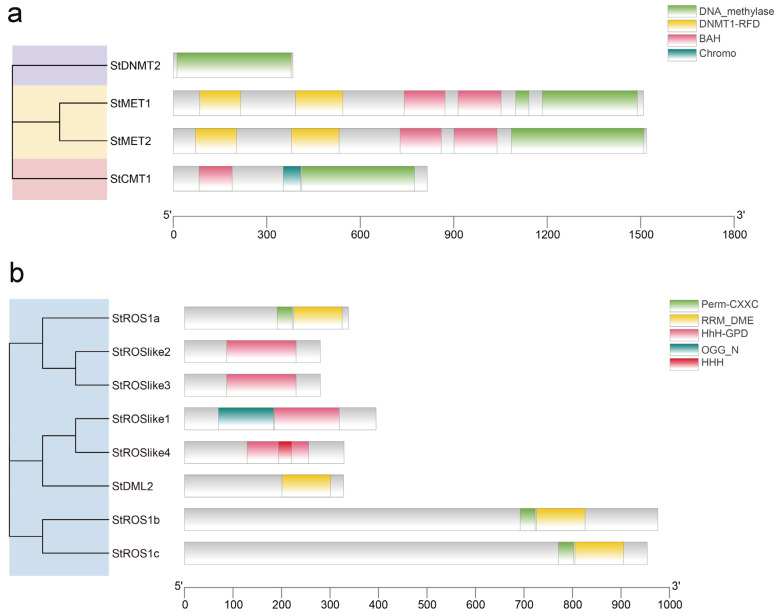
Conserved domain analysis of StC5-MTases and StdMTases. (**a**) Conserved domain of StC5-MTases. (**b**) Conserved domain of StdMTases.

To characterize structural features of StC5-MTases and StdMTases, conserved domains and gene structures were analyzed. The two MET proteins contained two replication foci-targeting domains (DNMT1-RFD), two bromo-adjacent homology (BAH) domains, and one or two DNA methylase domains. StCMT1 (CMT subfamily) contained a chromo domain, a BAH domain, and a DNA methylase domain, whereas StDNMT2 contained only a DNA methylase domain (Figure 2a). For StdMTases, StROS1a/1b/1c (ROS subfamily) carried an N-terminal RRM_DME domain and a C-terminal Perm-CXXC domain. All StROSlike proteins contained an HhH-GPD domain; StROSlike1 and StROSlike4 additionally contained an OGG_N domain and an HHH domain, respectively. StDML2 (DML subfamily) contained only an N-terminal RRM_DME domain (Figure 2b).

**Figure 3:**
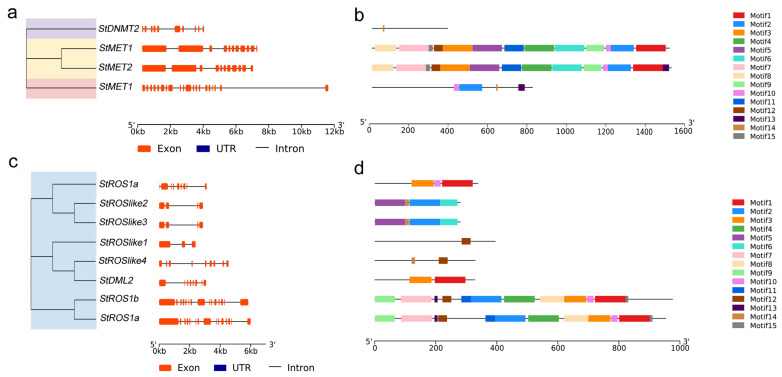
Phylogenetic relationships, gene structures, conserved motifs of StC5-MTases and StdMTases. (**a**) Phylogenetic relationships and gene structures of StC5-MTases. (**b**) Conserved motifs of StC5-MTases. (**c**) Phylogenetic relationships and gene structures of StdMTases. (**d**) Conserved motifs of StdMTases.

To further evaluate conservation and divergence within each family, conserved motifs were identified using Multiple Expectation Maximization for Motif Elicitation (MEME). Fifteen motifs were detected in each family. Motif lengths ranged from 7 to 150 amino acids in StC5-MTases and from 9 to 100 amino acids in StdMTases (Supplemental Table S2). StMET1 and StMET2 contained the largest number of motifs and included all motifs except motif 14, whereas StCMT1 contained four motifs (2, 10, 13, and 14) and StDNMT2 contained only motif 14 (Figure 3a). Among StdMTases, StROS1b and StROS1c contained all motifs except 5, 6, and 14, while StROSlike2 and StROSlike3 shared four motifs (2, 5, 6, and 14). StROSlike1, StROSlike4, StDML2, and StROS1a contained one (12), two (2 and 14), two (1 and 3), and three (1, 3, and 10) motifs, respectively (Figure 3b). Shared motif patterns likely reflect conserved functional modules within each subfamily.

Importantly, several motifs correspond to hallmark catalytic signatures reported for plant C5-MTases. For example, motif9 contains an FxGxG-like sequence consistent with SAM binding, motif10 includes the conserved PCQ catalytic signature, and motif2 contains the ENV motif, together supporting that StC5-MTases harbor the canonical cytosine-5 DNA methyltransferase catalytic core. For StdMTases, the conserved motif patterns are consistent with DEMETER/ROS1-type 5mC DNA glycosylases, in agreement with their characteristic domain composition (e.g., HhH-GPD and RRM_DME modules). However, motif-level functional assignments remain putative and require experimental validation.

#### 2.2.3. Chromosomal Distribution and Exon–Intron Structures of Genes Encoding StC5-MTases and StdMTases

StC5-MTase coding sequences contained 9–27 introns, whereas StdMTase genes contained 2–14 introns. *StMET1* had the most introns (27) and *StDNMT2* the fewest (9); *StMET2* and *StCMT1* contained 10 and 19 introns, respectively. Among StdMTases, *StROSlike2*/*StROSlike3*, *StROS1a*/*StROSlike4*, and *StROS1b*/*StROS1c* had the same intron numbers (3, 8, and 14, respectively), whereas *StROSlike1* and *StDML2* contained 2 and 7 introns, respectively. Exon–intron structures are summarized in Table 1 and Figure 3.

**Figure 4:**
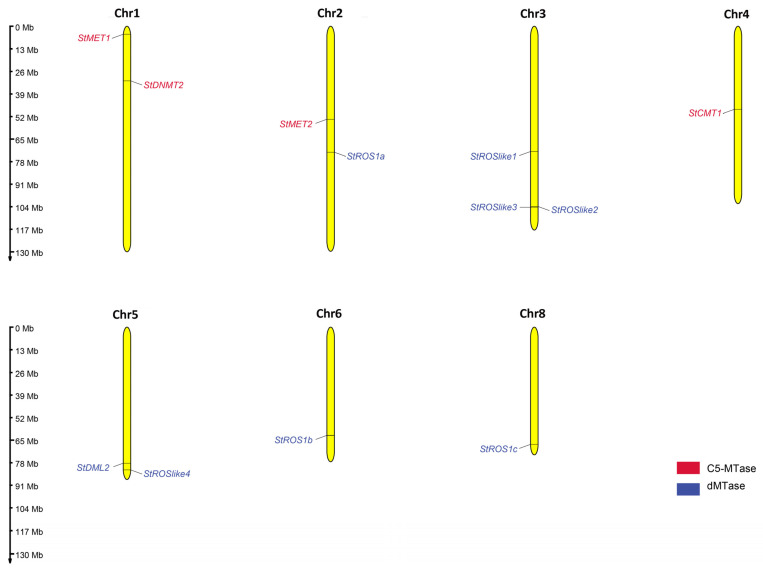
Chromosomal distribution of C5-MTase- and dMTase-encoding genes in *S. tonkinensis* genome.

The four StC5-MTase genes were located on chromosomes 1 (*StMET1* and *StDNMT2*), 2 (*StMET2*), and 4 (*StCMT1*). The eight StdMTase genes were distributed across chromosomes 2 (*StROS1a*), 3 (*StROSlike1/2/3*), 5 (*StDML2* and *StROSlike4*), 6 (*StROS1b*), and 8 (*StROS1c*) (Figure 4).

#### 2.2.4. Gene Duplication and Synteny Analysis of Genes Encoding StC5-MTases and StdMTases

**Figure 5:**
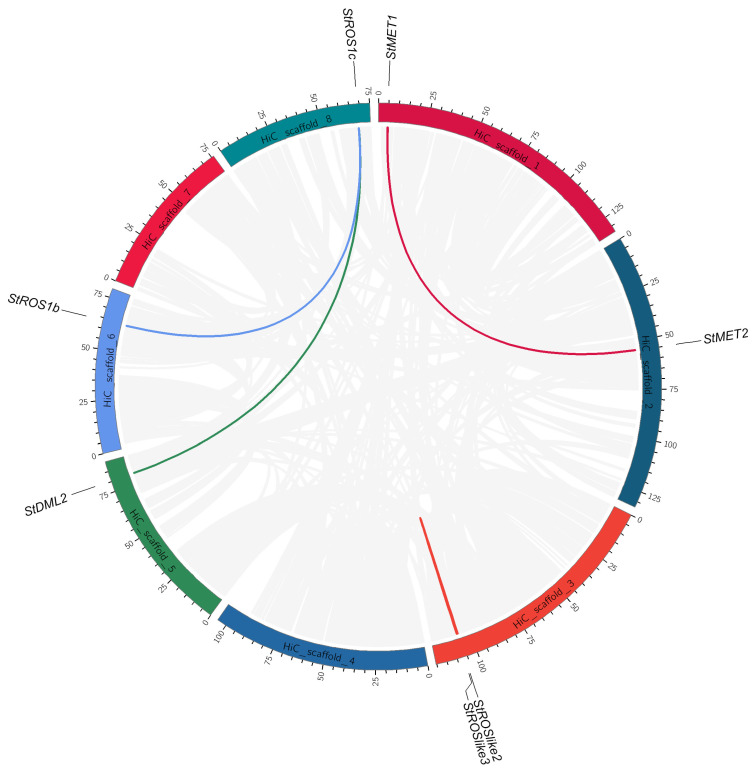
Collinearity analysis of the C5-MTase and dMTase genes in the *S. tonkinensis* genome. Gray lines indicate syntenic blocks across the genome. Segmental duplicated StC5-MTase and StdMTase gene pairs on different chromosomes highlighted and labeled in color.

To explore the evolutionary mechanisms shaping C5-MTase and dMTase families in *S. tonkinensis*, intra- and inter-species synteny analyses were conducted using MCScanX. Four duplicated gene pairs showed collinearity: *StMET1*/*StMET2*, *StROS1b*/*StROS1c*, and *StROS1c*/*StDML2*, *StROSlike2*/*StROSlike3* (Figure 5). The Ka/Ks ratios were <1.0 for three pairs, consistent with predominant purifying selection after duplication. Detailed Ka, Ks, and Ka/Ks values for all duplicated pairs are provided in Supplementary Table S3.

**Figure 6:**
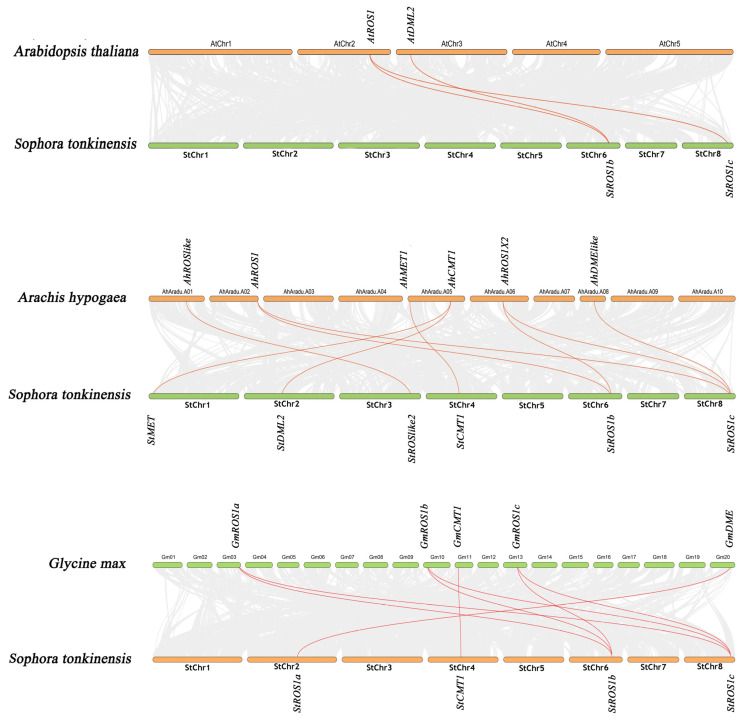
Collinearity synteny relationships of C5-MTase and dMTase genes between *S. tonkinensis* and three plant species (*G. max*, *A. hypogaea*, *A. thaliana*). The gray lines in the background indicate the collinear blocks within *S. tonkinensis* and other plant genomes.

Inter-species synteny analyses indicated that StC5-MTases and StdMTases had the highest conservation with *A. hypogaea* (nine orthologous pairs), followed by *G. max* (eight pairs) and *A. thaliana* (three pairs) (Figure 6), consistent with their close phylogenetic relationships. In *A. hypogaea*, two and three gene copies were orthologous to *StROS1b* and *StROS1c*, respectively. In addition, *StROS1b* had two orthologs in *A. thaliana* and three in *G. max*, whereas *StROS1c* had one ortholog in *A. thaliana* and three in *G. max*.

### 2.3. Promoter Cis-Elements and Stress-Responsive Expression of C5-MTases and dMTases

#### 2.3.1. Cis-Acting Regulatory Elements in the Promoter Regions of StC5-MTases and StdMTases

**Figure 7:**
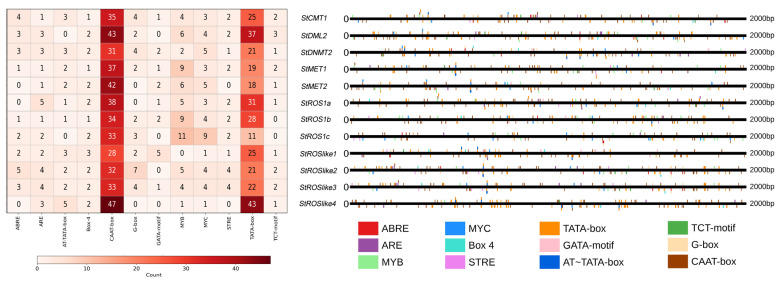
Cis-element analysis of C5-MTase and dMTase genes in *S. tonkinensis*.

Cis-acting regulatory elements (CREs) within promoters are key determinants of transcriptional regulation. To infer potential regulatory inputs for StC5-MTase and StdMTase genes, CREs in the 2 kb upstream regions were predicted using PlantCARE. All promoters contained core promoter motifs (e.g., TATA-box and CAAT-box) and multiple regulatory elements. Ten major CRE types were detected and grouped into light-responsive, stress-related, and phytohormone-responsive categories (Figure 7). Stress- and hormone-related motifs were broadly distributed, including ABRE (ABA-responsive) and ARE (anaerobic-responsive) elements. Light-responsive motifs (e.g., Box 4, G-box, and TCT-motif) were also prevalent: Box 4 occurred in all promoters, while G-box was absent only from *StMET1*, *StROS1a*, and *StROSlike4*. The TCT-motif was present in all promoters except *StROS1a* and *StROS1b*. Overall, these results suggest that StC5-MTase and StdMTase genes may be regulated by multiple environmental and hormonal cues in *S. tonkinensis*.

#### 2.3.2. Expression of C5-MTases and dMTases in *S. tonkinensis* Under Abiotic Stress

**Figure 8:**
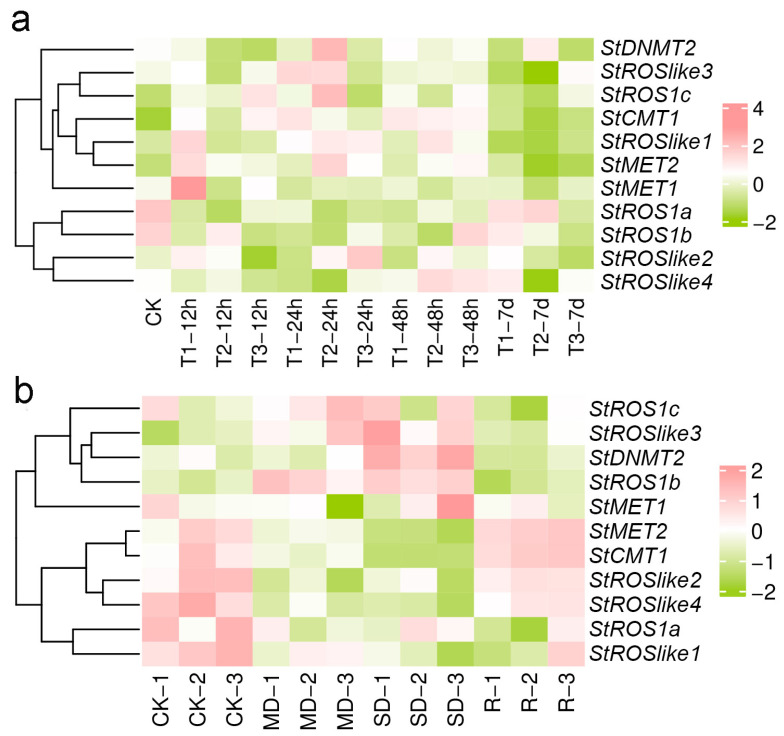
Heat map showing the expression patterns of StC5-MTase and StdMTase genes under abiotic stress. (**a**) Cd stress. (**b**) Drought and rewatering. Expression levels (Log2(TPM + 1)) are indicated by the color scale. CK, control; MD, moderate drought; SD, severe drought; R, rewatering. T1, 40 μM CdCl_2_; T2, 80 μM CdCl_2_; T3, 160 μM CdCl_2_.

Under Cd stress, transcripts of C5-MTase and dMTase responded dynamically in a time- and concentration-dependent manner (Figure 8a). During the early phase (12–48 h), distinct induction patterns were observed across treatments. Under T1, *StMET1* and *StROSlike1* were induced at 12 h, followed by *StROSlike3* at 24 h. Under T2, *StROS1c* and *StROSlike3* were induced at 24 h, whereas *StROSlike1* and *StROSlike4* increased at 48 h. Under T3, *StROS1c*, *StROSlike2*, and *StROS1b* were sequentially induced at 12, 24, and 48 h, respectively. Across treatments, *StCMT1* and *StMET2* showed sustained induction during 12–48 h. By 7 days, many early-responsive genes declined, *StROS1a* under T1 and T2 partially recovered toward CK (remaining slightly lower), whereas *StROS1b* was suppressed under high Cd (T3). *StDML2* was not detected, while *StDNMT2* remained relatively stable. Overall, these results indicate broad transcriptional shift of methylation-related genes during Cd stress.

Under drought and rehydration, *C5-MTases* and *dMTases* displayed stress-dependent patterns (Figure 8b). *StMET2* and *StCMT1* were progressively downregulated with increasing drought severity and rebounded after rewatering. In contrast, *StROS1b* and *StROSlike3* were induced by drought. *StROSlike2* and *StROSlike4* were strongly repressed under drought and recovered rapidly after rewatering, whereas *StROS1a* remained low after rewatering. *StDML2* again showed no detectable expression, while *StDNMT2* remained comparatively high across treatments. These results suggest coordinated yet gene-specific transcriptional response to water deficit and recovery.

**Figure 9:**
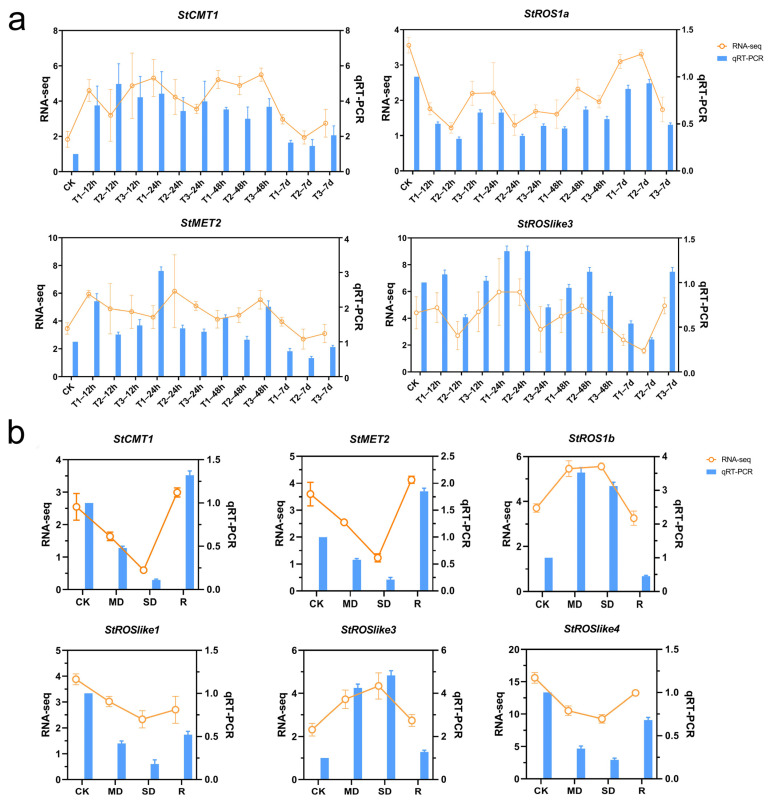
Validation of C5-MTase and dMTase gene expression patterns under abiotic stress. (**a**) C5-MTase and dMTase gene expression patterns under Cd stress. (**b**) C5-MTase and dMTase gene expression patterns under drought and rewatering conditions. Data from RNA-seq (TPM, left Y-axis) and qRT-PCR (2^−ΔΔCT^, right Y-axis) are shown for four genes in response to Cd and six genes in response to drought/rewatering.

For qRT-PCR validation, genes were selected based on (i) strong and reproducible RNA-seq responses, (ii) representative expression patterns (early vs. sustained; methyltransferase vs. demethylase), and (iii) biological relevance from phylogenetic classification and promoter CRE annotations. Genes with low/undetectable expression were not prioritized. For most genes, qRT-PCR trends were consistent with the RNA-seq profiles, supporting the reliability of the transcriptome data (Figure 9).

### 2.4. Overexpression of StCMT1 and StROSlike3 Enhances Abiotic Stress Tolerance in Transgenic Nicotiana benthamiana

**Figure 10:**
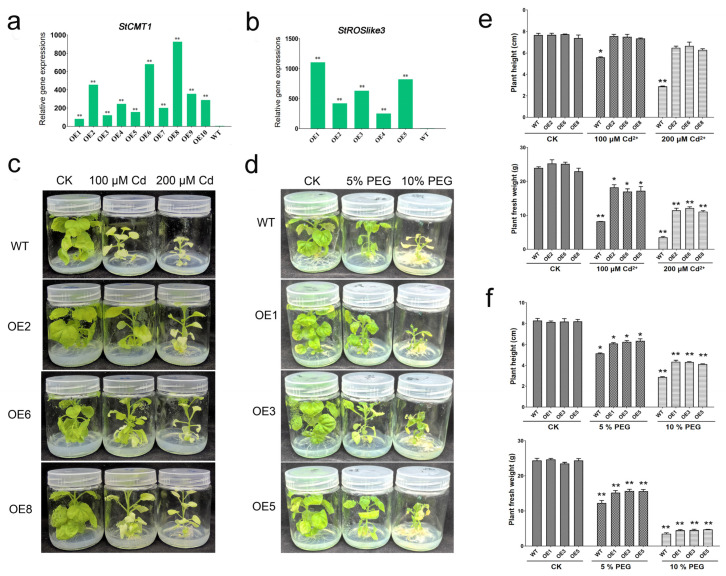
Phenotypic analysis of *Nicotiana benthamiana* overexpressing *StCMT1* or *StROSlike3*. (**a**,**b**) qRT-PCR analysis of gene expression. (**c**,**e**) Growth comparison between wild type and the three selected *StCMT1* transgenic lines under Cd stress. (**d**,**f**) Growth comparison between wild type and the three selected *StROSlike3* transgenic lines under PEG-induced drought stress. OE, overexpression line; WT, wild type. * *p* < 0.05, ** *p* < 0.01.

Based on the expression analyses (Figure 8 and Figure 9), *StCMT1* was selected for functional validation because it showed consistently higher transcript levels than CK across all Cd concentrations and time points. *StROSlike3* was prioritized because its expression increased progressively with drought severity and decreased after rehydration, indicating a clear water-status-dependent response pattern. Stable transgenic *Nicotiana benthamiana* lines constitutively expressing each gene were generated. Multiple independent lines were confirmed by PCR (Figure S3), and three lines with high transgene expression were selected by qRT-PCR for phenotyping (Figure 10a,b). In response to Cd exposure, the *StCMT1*-overexpression lines exhibited less growth inhibition than WT, maintaining higher plant height and whole-plant fresh weight at both 100 and 200 μM CdCl_2_ (Figure 10c,e). Likewise, under PEG-induced water deficit, *StROSlike3*-overexpressing lines sustained greater plant height and biomass than WT at both 5% and 10% PEG. Under the more severe 10% PEG treatment, WT plants showed pronounced leaf yellowing, whereas the transgenic lines retained partial leaf greenness and displayed milder chlorosis, consistent with enhanced drought tolerance (Figure 10d,f).

## 3. Discussion

DNA methylation provides mechanistic interface between environmental cues and the genome by enabling flexible regulation of gene expression during stress. Here, we present the first genome-wide characterization of C5-MTase and dMTase gene families in *S. tonkinensis*. We further show that these methylation-related enzymes display dynamic, stress-dependent transcriptional responses to Cd exposure and drought/rehydration, highlighting candidate regulators patterns that may contribute to stress acclimation.

### 3.1. Genomic and Bioinformatic Features of C5-MTase and dMTase Families in S. tonkinensis

We identified four C5-MTases in *S. tonkinensis* and assigned them to the MET, CMT, and DNMT subfamilies based on phylogeny and domain composition (Figure 1a and Figure 2a; Table 1). Their protein sizes were comparable to those reported in other plant species, including *S*. *tuberosum* [22]. The C5-MTase repertoire in *S. tonkinensis* is smaller than that reported for several other dicots (e.g., *A*. *chinensis*, *S. tuberosum*, *A*. *thaliana*, and *A*. *hypogaea*), suggesting a relatively compact family in this medicinal legume [15,16,20,22]. Notably, no DRM genes were detected, whereas DRM genes are present in multiple angiosperms, suggesting lineage-specific retention or loss. All StC5-MTases were predicted to localize to the nucleus, consistent with their expected roles in chromatin-associated methylation maintenance and establishment.

For dMTases, two subfamilies (DML and ROS) were represented in *S. tonkinensis* (Figure 1b and Figure 2b). Only one DML member was identified, whereas the ROS subfamily contained seven genes, indicating a relative expansion of ROS-like demethylases compared with some reference species [15,20,25]. Subcellular localization predictions suggested that most StdMTases are nuclear; however, WoLF PSORT predicted chloroplast targeting for StROS1a, StROSlike1, and StROSlike4, while Plant-mPLoc supported chloroplast localization only for StROSlike4 (Supplementary Table S1). Although these results are in silico and require experimental validation, the consensus candidate StROSlike4 (and other putative chloroplast-targeted proteins) may be linked to chloroplast-associated stress processes, given that chloroplasts are major sources of ROS under Cd stress and drought impairs photosynthesis. Consistently, these genes were transcriptionally responsive to Cd and drought/rehydration (Figure 8), supporting their potential involvement in stress responses.

Phylogenetic relationships revealed clear separation between dicots and monocots clades for both families (Figure 1), and *S. tonkinensis* genes were most closely related to homologs from the legume *A. hypogaea*, consistent with taxonomic affinity. Collinearity analysis identified four duplicated gene pairs (Figure 5), indicating that gene duplication has contributed to family diversification. Ka/Ks ratios were <1.0 for three duplicated pairs, consistent with predominant purifying selection after duplication. The *StROSlike2*/*StROSlike3* pair exhibited an exceptionally low Ks, which can destabilize Ka/Ks estimates for very recent duplicates; thus, this pair was interpreted cautiously and not used as evidence for positive selection (Supplementary Table S3). While synonymous substitutions (Ks) can, in principle, be used to approximate divergence time (T = Ks/2r), reliable time estimates require a species-appropriate neutral substitution rate, which is currently unavailable for *S. tonkinensis*.

Promoter scanning identified multiple stress- and hormone-related CREs in StC5-MTase and StdMTase promoters (Figure 7), which is consistent with their stress-responsive transcription. For example, the sustained induction of *StCMT1* and *StMET2* under Cd and drought, together with their decline after rehydration, may be partly associated with the presence of ABRE and other stress-related CREs, suggesting potential responsiveness to ABA/stress signaling. However, this CRE survey is descriptive and does not by itself demonstrate statistical enrichment or functional activity; experimental promoter assays and/or genome-wide background comparisons will be required for further validation.

### 3.2. Transcriptional Responses of StC5-MTases and StdMTases Under Cd Stress

Cd contamination is a major constraint on *S. tonkinensis* growth and productivity [29,30]. In plants, Cd stress is frequently accompanied by ROS accumulation, hormonal reprogramming, and broad transcriptional remodeling, and methylation-related enzymes are often transcriptionally responsive under such conditions [33,34,35]. In our study, Cd exposure triggered rapid and concentration-dependent transcriptional changes in *StC5-MTases* and *StdMTases* (Figure 8a). Under high Cd (T3), *StROS1c*, *StROSlike2*, and *StROS1b* were induced sequentially at 12, 24, and 48 h, respectively, indicating temporally staged activation under severe stress. In parallel, *StCMT1* and *StMET2* were consistently upregulated across early to mid stages, suggesting that these methylation “writer” genes are transcriptionally responsive during Cd exposure. At 7 days, *StROS1a* was induced under T1 and T2 as many early-responsive genes declined, consistent with a transition from acute responses toward longer-term adjustment. Together, these results indicate coordinated transcriptional engagement of methylation-related genes during Cd stress, although transcript changes alone do not directly demonstrate corresponding changes in DNA methylation patterns.

### 3.3. Transcriptional Responses of StC5-MTases and StdMTases Under Drought and Rehydration

Drought is another major factor limiting *S. tonkinensis* growth and yield [31,32]. Drought responses involve interconnected physiological adjustments and large-scale transcriptional reprogramming, and DNA methylation has been implicated as one regulatory layer in multiple plant species [36,37,38,39]. In *S. tonkinensis*, drought elicited a multi-phase transcriptional pattern in methylation-related genes (Figure 8b). *StCMT1* and *StMET2* were progressively suppressed as drought severity increased and then rebounded after rewatering, consistent with stress- and recovery-associated transcriptional regulation of methylation “writer” genes. In contrast, dMTases such as *StROS1b* and *StROSlike3* increased with drought severity, supporting their potential involvement in drought-responsive regulatory programs. *StROSlike4* decreased progressively under drought and recovered after rewatering, suggesting sensitivity to water status. While these expression patterns are consistent with roles for methylation machinery in drought responses, direct methylation measurements will be required to determine whether they translate into locus-specific or genome-wide methylation changes.

### 3.4. Shared Versus Stress-Specific Responses and Implications of Functional Validation

Cadmium toxicity and drought trigger partially overlapping but largely distinct stress-response pathways in plants. Cd stress is typically associated with metal uptake and transport, thiol-based chelation and vacuolar sequestration (e.g., glutathione/phytochelatins), and pronounced oxidative stress that requires reinforced antioxidant capacity and redox homeostasis [40]. By contrast, drought responses are more tightly linked to ABA biosynthesis and signaling, stomatal regulation, osmotic adjustment, and protection of photosynthetic performance [41,42]. In addition, recent epigenomic studies increasingly integrate methylome profiling with transcriptomics to dissect stress-induced regulatory remodeling under drought and heavy-metal exposure [43,44]. Against this background, comparing C5-MTase and dMTase transcriptional responses under Cd versus drought/rehydration provides a useful first step to distinguish shared versus stress-preferential regulatory programs in *S. tonkinensis*.

Comparative analysis across Cd and drought/rehydration revealed both shared and stress-preferential expression patterns. *StCMT1* and *StMET2* displayed pronounced transcriptional plasticity, remaining elevated during Cd exposure while showing recovery-associated rebound after drought, indicating responsiveness to both stress and relief phases. Importantly, *StROS1b* and *StROSlike3* were induced early under both stresses, consistent with roles in early stress-responsive regulatory programs. Several genes exhibited more stress-preferential behavior: *StROSlike4* responded strongly to drought, whereas *StROS1a* was induced during longer-term Cd exposure but remained low after drought recovery, suggesting functional specialization. The relatively stable expression of *StDNMT2* is consistent with a housekeeping-like role, while the consistently low/undetectable expression of *StDML2* may reflect tissue specificity or restricted expression. Functional assays in transgenic *N*. *benthamiana* provide phenotypic evidence that manipulating *StCMT1* or *StROSlike3* can influence plant performance under Cd or PEG-simulated drought stress (Figure 10). Future work should quantify physiological indicators in the overexpression lines, such as H_2_O_2_/O^2−^ accumulation, MDA content, electrolyte leakage, antioxidant enzyme activities (SOD, CAT, APX), proline content, chlorophyll level/photosynthetic performance, and Cd accumulation, to better connect the observed phenotypes with specific tolerance mechanisms [45,46]. Similar readouts are routinely used to substantiate improved Cd or drought tolerance in transgenic *Nicotiana* systems [47,48,49].

In addition, an in silico protein–protein interaction analysis predicted putative associations among StC5-MTase and StdMTase proteins, with *StCMT1* showing potential links to *StMET2*, *StMET1*, and *StDNMT2* (Figure S4). These predicted interactions provide testable hypotheses but require experimental validation. Finally, we emphasize that changes in the transcript levels of *StC5-MTases* and *StdMTases* do not directly demonstrate altered DNA methylation states at specific loci or genome-wide. Without methylome-level measurements (e.g., WGBS/BS-seq) or locus-specific methylation assays, causality between gene expression and methylation changes cannot be inferred. Future work integrating methylome profiling with transcriptome data will be required to test whether the observed transcriptional responses translate into stress-induced methylation remodeling. Moreover, transcript abundance does not necessarily translate into protein abundance or enzymatic activity, and post-transcriptional and post-translational regulation (e.g., alternative splicing, miRNA-mediated regulation, translational control, protein stability/modification) as well as indirect stress effects (growth inhibition, hormonal reprogramming, or tissue composition changes) may contribute to the observed patterns. Therefore, mechanistic interpretation should be made cautiously and will benefit from future analyses at the protein/activity level.

## 4. Materials and Methods

### 4.1. Identification of C5-MTase and dMTase Genes

Protein sequences of C5-MTase and dMTase family members from *Arabidopsis thaliana* and *Arachis hypogaea* were used as reference queries. To identify putative C5-MTase and dMTase genes in *S. tonkinensis*, hidden Markov models (HMMs) were built and searched using HMMER 3.0 [50] against all predicted *S. tonkinensis* protein sequences. In parallel, BLASTP (ncbi-blast-2.10.1+) searches were performed using *A. thaliana* and *A. hypogaea* C5-MTase and dMTase proteins as queries with an E-value cutoff of 1 × 10^−5^. Candidates from both approaches were merged and subjected to domain annotation with PfamScan (v1.6) against the Pfam-A database (v33.1) [51,52]. Sequences containing PF00145 were retained as candidate C5-MTase genes, whereas sequences containing PF00730 (HhH-GPD) and/or PF15628 (RRM_DME) were retained as candidate DEMETER-like DNA demethylase (dMTase) genes. Specifically, PF00145 corresponds to the conserved C5 cytosine-specific DNA methyltransferase catalytic domain, while PF00730 and PF15628 are characteristic domains of plant DEMETER-like DNA demethylases and were used to distinguish dMTase candidates.

### 4.2. Phylogenetic Analysis

Full-length amino-acid sequences of *C5-MTases* and *dMTases* from *S. tonkinensis*, *A*. *hypogaea*, *A*. *thaliana*, *G*. *max*, *O*. *sativa*, and *Z*. *mays* were aligned using MAFFT v7.427 with default parameters. These five species were chosen to represent well-annotated legumes (peanut and soybean), a dicot model (Arabidopsis), and two representative monocots (rice and maize), providing a broad evolutionary framework for subfamily classification. NJ phylogenetic trees were constructed in MEGA 11 [53], with 1000 bootstrap replicates. The p-distance model was used, and gaps were treated using partial deletion. Trees were visualized and annotated using the Interactive Tree of Life (iTOL v5) online tool [54]. To validate the NJ-based inference, an ML tree was additionally reconstructed using IQ-TREE 2 with 1000 bootstrap replicates under the best-fit substitution model selected by ModelFinder as implemented in IQ-TREE 2.

### 4.3. Analysis of Conserved Motifs, Subcellular Localization, and Physicochemical Properties

Conserved motifs in the StC5-MTase and StdMTase proteins were identified with the MEME suite (Multiple Expectation Maximization for Motif Elicitation; http://meme-suite.org/meme, accessed on 5 November 2025) [55]. Subcellular localization was predicted using WoLF PSORT (https://wolfpsort.hgc.jp/, accessed on 7 November 2025) and Plant-PLoc (http://www.csbio.sjtu.edu.cn/bioinf/plant/, accessed on 12 November 2025). Physicochemical properties, including molecular weight (MW), theoretical isoelectric point (pI), amino-acid composition, instability index (II), aliphatic index (AI), and grand average of hydropathicity (GRAVY), were computed using ProtParam (ExPASy) (http://web.expasy.org/protparam, accessed on 15 November 2025).

### 4.4. Analysis of Gene Structure and Chromosomal Location

Gene structures (exon–intron organization) and chromosomal coordinates of StC5-MTase and StdMTase genes were obtained from the *S. tonkinensis* genome annotation (GFF) (GSA, PRJCA053979). Gene-structure diagrams were generated using the Gene Structure Display Server (GSDS; http://gsds.gao-lab.org/, accessed on 17 November 2025) [56], and chromosomal distribution was visualized with MapGene2Chromosome v2.0 (MG2C, http://mg2c.iask.in/mg2c_v2.0/, accessed on 17 November 2025) [57].

### 4.5. Collinearity and Synteny Analysis

Intra- and inter-species collinearity analyses of C5-MTase and dMTase genes were performed using MCScanX (v2012), and results were visualized with TBtools (v2.096) [58,59]. Non-synonymous (Ka) and synonymous (Ks) substitution rates for duplicated gene pairs were calculated using KaKs_Calculator [60]. The Ka/Ks ratio was used as an indicator of selective pressure acting on the coding sequences.

### 4.6. Cis-Acting Element Analysis

For each StC5-MTase and StdMTase gene, the 2000 bp sequence upstream of the transcription start site was retrieved using TBtools [47]. Putative cis-acting regulatory elements within these promoter regions were identified using PlantCARE (https://bioinformatics.psb.ugent.be/webtools/plantcare/html/, accessed on 25 November 2025) [61].

### 4.7. Prediction of Protein Interaction Networks

Protein–protein interaction networks for the C5-MTase and dMTase families were predicted using the STRING database (https://cn.string-db.org/, accessed on 27 November 2025), with *A. thaliana* set as the reference organism and a minimum interaction confidence score of 0.40.

### 4.8. Expression Analysis of C5-MTase and dMTase Genes Under Cd and Drought Stress

Uniform *S. tonkinensis* seedlings (4-week-old) were used in this study. For each treatment, nine seedlings were used per replicate, and three independent biological replicates were performed. Cd stress was applied in a hydroponic system, whereas drought and subsequent rehydration treatments were conducted using soil-grown (potted) seedlings, as described below.

Seedlings of similar size were transferred to 1/2-strength Hoagland nutrient solution and acclimated for one week. Cd stress was then imposed by adding CdCl_2_ to the nutrient solution at final concentrations of 0, 40, 80, or 160 μM. Root tissues were chosen for transcriptomic analysis because they are the primary organs for Cd uptake. Root tissues were collected at 0, 12, 24, and 48 h, and at 7 days after treatment, immediately frozen in liquid nitrogen, and stored at −80 °C for subsequent RNA extraction.

For drought experiments, seedlings were grown in pots, and soil water status was controlled based on available soil water (ASW). Drought stress was imposed by controlling soil water availability. As with Cd treatment, roots were selected for analysis due to their primary role in perceiving drought stress and initiating early responses to water deficit. The target ASW ranges were maintained by soil moisture meter and watering to the required level throughout the treatment period. Control plants (CK) were maintained at 75–80% ASW for 15 days. Mild drought (MD) treatment was maintained at 55–60% ASW for 15 days, and severe drought (SD) treatment was maintained at 30–35% ASW for 15 days. For rehydration (R), SD-treated plants were rewatered to restore soil water content to 75–80% ASW and were sampled 7 days after rewatering. Roots were harvested at the corresponding stages, immediately frozen in liquid nitrogen, and stored at −80 °C until use.

Total RNA was extracted using TRIzol reagent (Invitrogen, Thermo Fisher Scientific, Waltham, MA, USA) according to the manufacturer’s instructions. RNA-seq libraries were prepared from 1 μg total RNA using the Illumina Stranded mRNA Prep, Ligation Kit (Illumina, San Diego, CA, USA) and sequenced on the NovaSeq X Plus platform (Illumina, San Diego, CA, USA) as 150 bp paired-end reads. Transcriptomic data were processed using the Omicsmart online platform (http://www.omicsmart.com, accessed on 3 November 2025). Expression levels of C5-MTase and dMTase genes were retrieved and normalized as transcripts per million (TPM) (Supplemental Tables S3 and S4). For clustering, log2(TPM + 1)-transformed values were subjected to hierarchical clustering in Cluster 3.0 and visualized in Java TreeView (v1.0).

### 4.9. qRT-PCR Validation of Gene Expression

For qRT-PCR validation, the total RNA samples described in Section 4.8 were used. First-strand cDNA was synthesized with the HiScript III RT SuperMix for qPCR (Vazyme, Nanjing, China). Gene-specific primers were designed using Primer Premier 5.0 (Supplemental Table S5). qRT-PCR was performed following the method of Liang et al. [62]. Reaction specificity was confirmed by melting-curve analysis, and relative gene expression was normalized to the reference gene *Actin* and calculated using the 2^−ΔΔCT^ method. Data are presented as mean ± SD from at least three independent experiments.

### 4.10. Heterologous Overexpression and Stress Phenotyping in N. benthamiana

To functionally validate *StCMT1* and *StROSlike3*, their full-length coding sequences were amplified from *S. tonkinensis* cDNA using gene-specific primers (Supplemental Table S6). The coding sequences of *StCMT1* and *StROSlike3* were cloned into the plant expression vector pHK-35S via Eco31I/BsaI sites, generating Pro35S:StCMT1 and Pro35S:*StROSlike3* constructs, respectively. Each construct was introduced into *Agrobacterium tumefaciens* strain GV3101 and used to transform *N. benthamiana* via the leaf disc method [63]. Primary transformants (T0) were screened by PCR using primer pairs that spanned the transgene and the vector (Supplemental Table S6). Positive plants were grown to maturity, and T1 seeds were harvested. Transgenic T1 seedlings were selected on MS medium supplemented with 50 mg/L hygromycin B and confirmed by PCR. For each construct, three independent transgenic lines with high transgene expression levels (as determined by qRT-PCR) were selected for subsequent stress assays. Differences between two groups were assessed by Student’s t-test, while comparisons across multiple conditions were analyzed by one-way ANOVA. Statistical significance is defined as * *p* < 0.05 or ** *p* < 0.01. *StCMT1*-overexpressing lines were subjected to Cd stress on MS medium containing 100 or 200 μM CdCl_2_, while *StROSlike3*-overexpressing lines were subjected to osmotic stress on MS medium containing 5% or 10% (*w*/*v*) PEG 6000 to simulate drought. Wild-type plants were included as controls. All plants were grown under controlled conditions for 20 days and then photographed to record growth phenotypes.

## 5. Conclusions

In conclusion, this study presents the first comprehensive analysis of C5-MTase and dMTase gene families in *S. tonkinensis*, providing insights into their evolutionary dynamics and stress-responsive expression profiles. Through functional validation in *N. benthamiana*, we demonstrated that *StCMT1* enhances Cd tolerance, while *StROSlike3* improves drought resistance, linking these epigenetic regulators to distinct stress adaptation pathways. These findings lay the groundwork for further studies on DNA methylation in stress responses and offer potential targets for epigenetics-based breeding to enhance stress resilience in this important medicinal plant.

## Figures and Tables

**Table 1:** Basic information of cytosine-5 DNA methyltransferases (C5-MTase) and DNA demethylases (dMTase) genes in *S. tonkinensis*.

Gene Name	Gene ID	CDS Length (bp)			Protein					
			Exon Number	Intron Number	Number of aa	MW (kDa)	pI	II	AI	GRAVY
C5-MTases										
CMT1	evm.TU.HiC_scaffold_4.2836	2448	20	19	815	92.04	5.47	39.4	76.98	0.052
MET1	evm.TU.HiC_scaffold_1.250	4530	28	27	1509	170.90	5.78	46.22	76.13	0.503
MET2	evm.TU.HiC_scaffold_2.4148	4560	11	10	1519	170.94	5.74	41.85	75.37	0.502
DNMT2	evm.TU.HiC_scaffold_1.2492	1155	10	9	384	43.73	6.23	50.11	78.75	0.366
dMTases										
DML2	evm.TU.HiC_scaffold_5.2915	987	8	7	328	37.02	4.70	51.16	71.04	0.577
ROS1a	evm.TU.HiC_scaffold_2.4917	1017	9	8	338	38.59	5.23	49.26	73.58	0.667
ROS1b	evm.TU.HiC_scaffold_6.1625	2931	15	14	976	109.33	5.63	48.98	70.45	0.735
ROS1c	evm.TU.HiC_scaffold_8.2263	2865	15	14	954	107.45	5.48	49.47	76.29	0.584
ROSlike1	evm.TU.HiC_scaffold_3.2420	1188	3	2	395	43.37	9.66	45.84	86.96	0.258
ROSlike2	evm.TU.HiC_scaffold_3.5115	843	4	3	280	31.67	9.15	37.78	83.57	0.620
ROSlike3	evm.TU.HiC_scaffold_3.5174	876	4	3	291	33.62	9.24	39.39	83.57	0.595
ROSlike4	evm.TU.HiC_scaffold_5.3220	990	9	8	329	36.61	9.32	44.8	88.6	0.301

MW, molecular weight; pI, isoelectric point; aa, amino acid; II, instability index; AI, Aliphatic index; GRAVY, grand average of hydrophobicity.

## Data Availability

All relevant data are within the manuscript and its Supplementary Files.

## Supplementary Materials

The following supporting information can be downloaded at: https://www.mdpi.com/article/10.3390/plants15030396/s1. Figure S1. Sequence similarity of *C5-MTases* and *dMTases* in *S. tonkinensis*. The colored bar indicates the correlation of two proteins. Red represents a high correlation; yellow represents a low correlation; blue represents no correlation analysis on these proteins. Figure S2. Phylogenetic tree of *S. tonkinensis* inferred by Maximum Likelihood (ML) method. (a) ML tree of the StC5-MTases. (b) ML tree of the StdMTases. Numbers at nodes represent bootstrap support values from 1000 replicates. Figure S3. Potential protein–protein interaction network of StC5-MTases and StdMTases. Figure S4. Identification of positive transgenic lines via PCR. (a) PCR analysis for identifying *StCMT1* transgenic plants. (b) PCR analysis for identifying *StROSlike3* transgenic plants. Table S1. Subcellular localization prediction of C5-MTases and dMTases from *S. tonkinensis*. Table S2. Conserved Motif Composition in C5-MTases and dMTases from *S. tonkinensis*. Table S3. Ka, Ks, and Ka/Ks values for all duplicated pairs. Table S4. Expression levels (TPM values) of *C5-MTases* and *dMTases* in *S. tonkinensis* under drought and rewatering conditions. Table S5. Expression levels (TPM values) of *C5-MTases* and *dMTases* in *S. tonkinensis* under Cd stress. Table S6. Primers used for the qRT-PCR of C5-MTases and dMTases in *S. tonkinensis*. Table S7. Primers used for vector construction and transgenic plant detection.
