# Supplementary figures and images for: Genome-Wide Identification and Functional Analysis of DNA Methylation-Related Genes in Sophora tonkinensis Under Cadmium and Drought Stress
Source: Plants (Basel). 2026 Jan 28;15(3):396. doi: 10.3390/plants15030396 (PMC12899016; doi:10.3390/plants15030396)

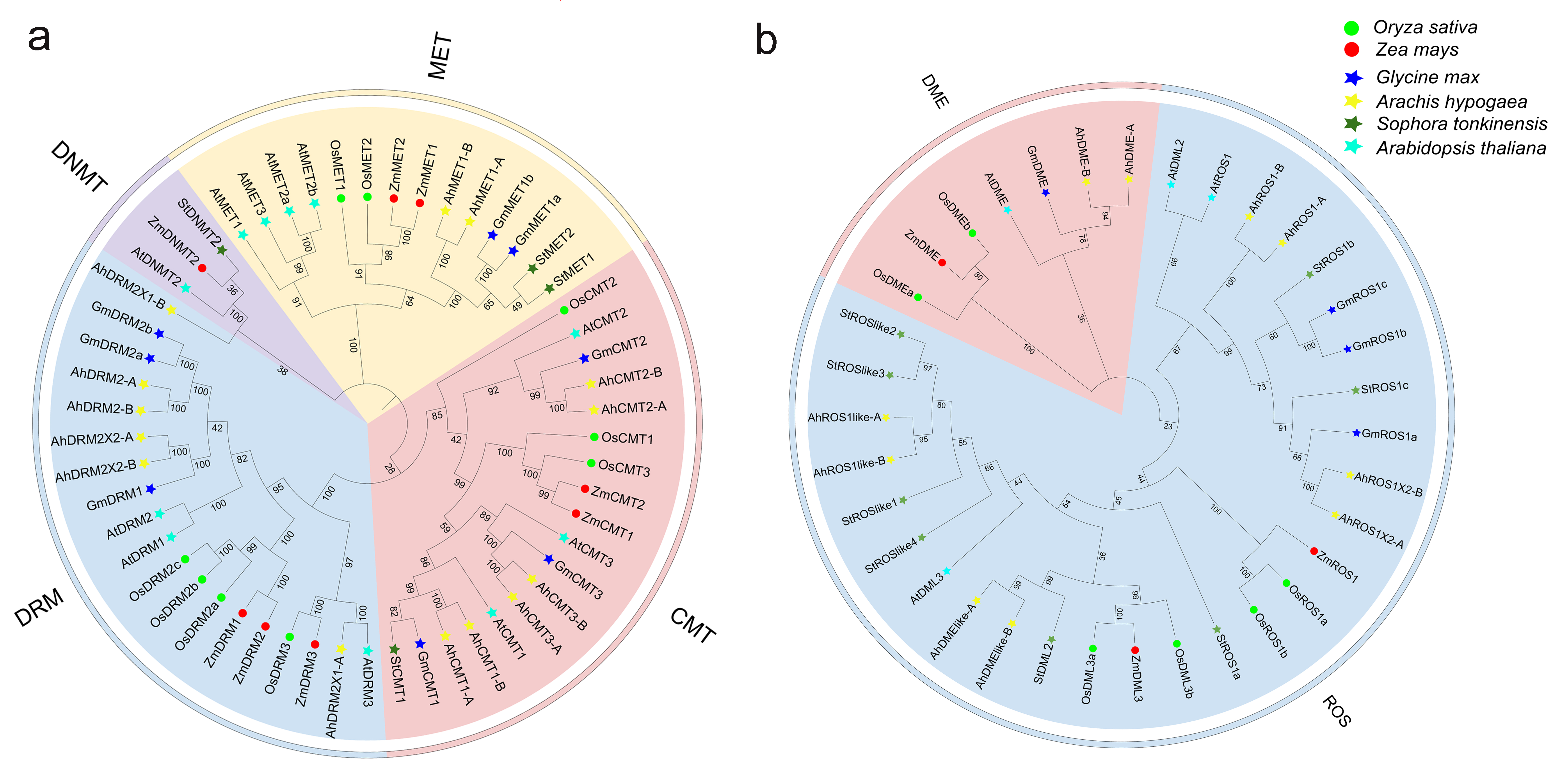

Supplement: Supplementary file 1 [file plants-15-00396-s001.zip › Figure S2.jpg]

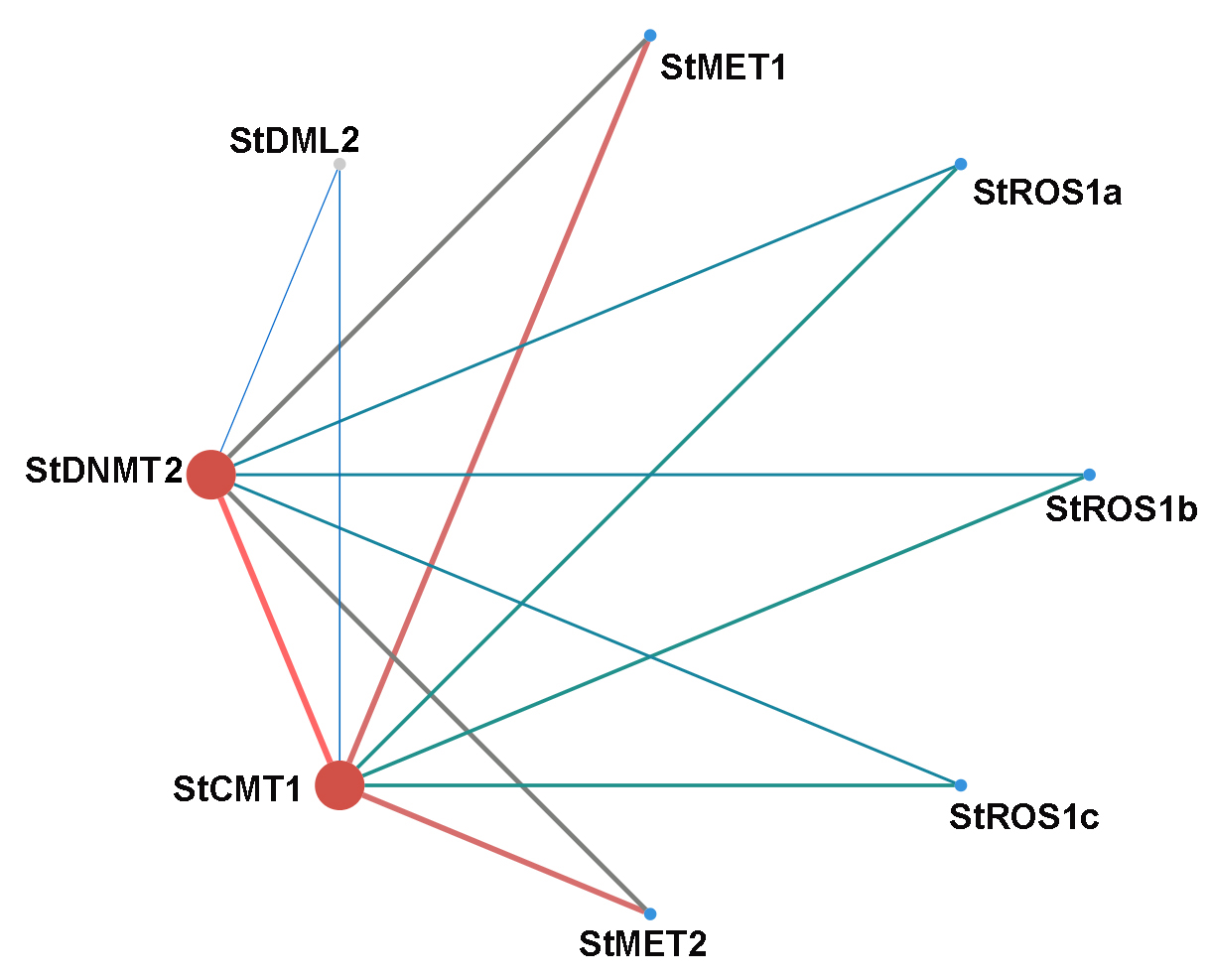

Supplement: Supplementary file 1 [file plants-15-00396-s001.zip › Figure S3.jpg]

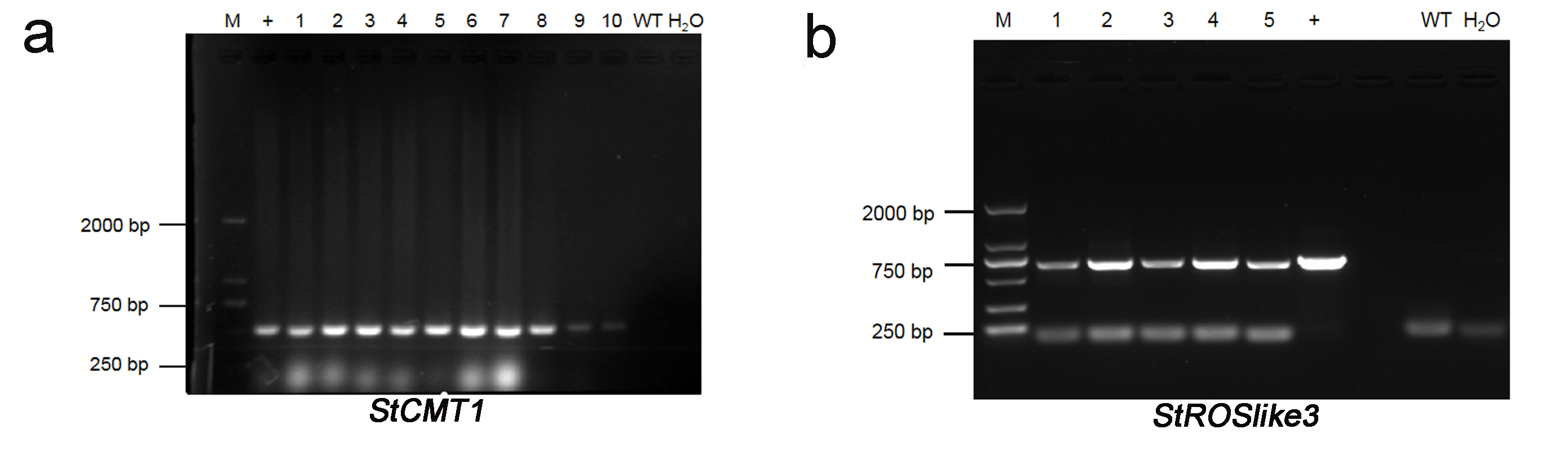

Supplement: Supplementary file 1 [file plants-15-00396-s001.zip › Figure S4.jpg]

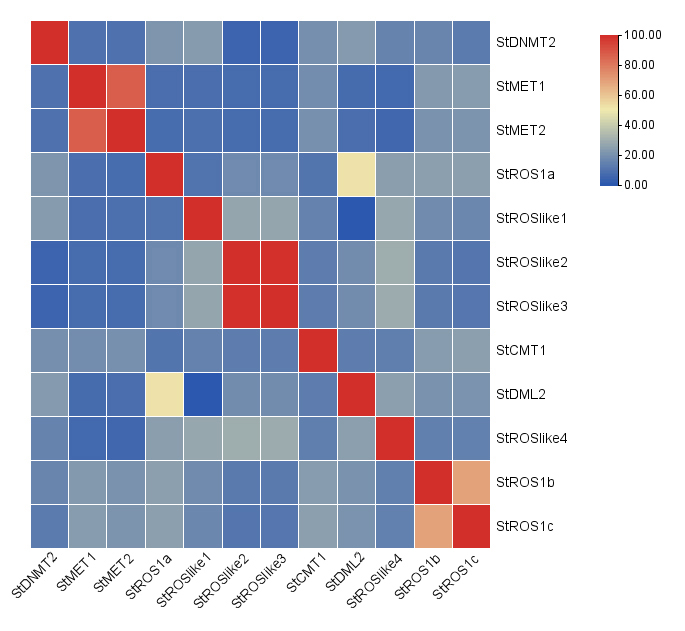

Supplement: Supplementary file 1 [file plants-15-00396-s001.zip › Figure S1.jpg]
